# Acculturative stress and achievement motivation: the moderating role of immigrant mothers’ Korean proficiency in South Korean multicultural adolescents

**DOI:** 10.3389/fpsyg.2025.1652737

**Published:** 2025-10-01

**Authors:** Yuanying Jin, Hyun Seon Ahn

**Affiliations:** ^1^Department of Education, College of Humanities, Sejong University, Seoul, Republic of Korea; ^2^Graduate School of Education, Korea University, Seoul, Republic of Korea

**Keywords:** acculturative stress, achievement motivation, immigrant mothers, Korean proficiency, multicultural youth panel survey

## Abstract

**Introduction:**

This study explores the impact of acculturative stress, mothers’ Korean language proficiency, and their combined effect on the achievement motivation of multicultural students in South Korea.

**Methods:**

Through hierarchical multiple regression analysis of data from 1,287 ninth-grade multicultural adolescents from the Multicultural Youth Panel Survey (MAPS), the relationships among these variables were examined.

**Results:**

The findings show that high acculturative stress is linked to lower achievement motivation. Notably, this decline is more significant when students perceive their mothers’ Korean language proficiency to be higher.

**Discussion:**

These findings emphasize the need to critically reassess the effectiveness of current multicultural support policies, which are primarily centered on improving mothers’ language proficiency, to better ensure their effectiveness in addressing targeted challenges and achieving meaningful outcomes. Further research is needed to delve deeper into the dynamics of achievement motivation under conditions of high acculturative stress.

## Introduction

Over the recent decades, South Korea has undergone significant demographic shifts, characterized by a notable increase in its multicultural population, driven primarily by international marriages. In 2022, South Korea recorded 16,666 international marriages, a 27.2% increase from the previous year, representing 8.7% of total marriages (Statistic Korea, 2023). These unions, predominantly involving Korean husbands and immigrant wives, constituted 80.3% of international marriages (Ministry of Justice, 2024), with a significant proportion of immigrant wives originating from Vietnam (27.6%), China (19%), and Thailand (16.1%) (Statistic Korea, 2023). As a direct result, multicultural families are now an integral part of Korean society, with their children making up 3.5% (181,178) of the student population – a significant increase from 0.7% in 2012 (Statistic Korea, 2024). However, despite their growing numbers, students from multicultural families face critical educational disparities, including a lower college entrance rate of 40.5% compared to the national average of 71.5% (E-Daily, 2023), and a higher middle school dropout rate of 0.78%, compared to 0.54% for the overall student population (E-Daily, 2023). These disparities signal the urgent need for targeted interventions aimed at supporting the educational attainment and social integration of multicultural students in South Korea.

Central to addressing these educational disparities is the concept of achievement motivation, which is recognized as a key determinant of academic success. Achievement motivation refers to the interplay between energization, which drives individuals to pursue competence-related behaviors, and the direction of these efforts toward goal orientation (Elliot and Sommet, 2023). As such, numerous studies have established a direct correlation between high levels of achievement motivation and positive academic outcomes (Abd-El-Fattah and Patrick, 2011; Diseth and Kobbeltvedt, 2010; Park, 2017; Yeon and Choi, 2020). In the case of multicultural students in South Korea, however, achievement motivation is often undermined by a variety of socio-cultural factors, including acculturative stress, low self-esteem, limited Korean language proficiency, parenting effectiveness, a lack of social support and teacher-student relationship (Choi, 2022; Choi et al., 2023; Lee, 2019; Park, 2017; Park and Son, 2023). While these factors have been individually studied, the extent to which they interact to impact the achievement motivation of multicultural students in the Korean educational context requires further exploration.

Among the various factors influencing achievement motivation, acculturative stress has emerged as a critical variable that significantly affects the educational experiences of multicultural students. Acculturative stress is defined as the psychological strain experienced by individuals attempting to adapt to a new or dominant culture (Berry, 1997), and it is especially pronounced in societies like South Korea, where collectivist values heighten the pressure to conform (Shin et al., 2023). Research indicates that acculturative stress is strongly associated with negative outcomes, such as depression, suicidal ideation, substance use, social withdrawal, and impaired school adjustment (Hovey, 2000; Park and Shin, 2022; Shin and Stephenson, 2024; Soufi Amlashi et al., 2024; Yoon et al., 2020). For multicultural students in South Korea, these challenges not only hinder their academic performance but also erode their overall achievement motivation (Lee et al., 2020; Park and Son, 2023). Addressing acculturative stress, therefore, is essential for improving both the educational attainment and the emotional well-being of multicultural students in South Korea.

An important, yet underexplored, factor that may alleviate the impact of acculturative stress is the Korean language proficiency of immigrant mothers. Despite government policies promoting bilingualism (Ministry of Gender Equality and Family, 2023), Korean remains the dominant language within multicultural households, with many immigrant mothers prioritizing fluency in Korean as essential for their children’s success in both academic and social domains (Ministry of Gender Equality and Family, 2022). In her qualitative study, Park (2019) found that immigrant mothers view Korean proficiency not only as a crucial tool for supporting their children’s education but also as integral to their own sense of identity and role fulfilment as “good Korean mothers.” However, when mothers perceive their language skills to be inadequate, they often experience feelings of guilt and self-blame, further intensifying their efforts to master the language. This linguistic emphasis reflects the broader societal reality in South Korea, where multicultural children are at greater risk of exclusion and marginalization (Oh, 2014). Consequently, a mother’s Korean proficiency becomes a pivotal factor in her ability to engage meaningfully in her children’s academic development and socialization processes.

It is widely recognized that immigrant mothers’ proficiency in the Korean language plays a pivotal role in shaping their children’s developmental outcomes (Park, 2019) with empirical evidence from prior studies highlighting significant associations between immigrant mothers’ proficiency in Korean language skills and their children’s language development (Woo et al., 2009), increased self-esteem (Park, 2010), and improved parenting practices (Jung, 2013). However, there is limited empirical research examining the direct impact of mothers’ Korean language proficiency on children’s achievement motivation, leaving this critical area underexplored. Maternal involvement, particularly in the form of responsiveness and engagement, has been shown to positively influence children’s socio-emotional development, including their social interaction, initiative, and self-regulation (Lerner and Grolnick, 2020; Park, 2017). It is, therefore, reasonable to hypothesize that mothers with higher proficiency in Korean may be better equipped to navigate the school system, communicate effectively with teachers, and support their children’s learning. Understanding the relationship between maternal language proficiency and children’s academic achievement is, therefore, essential in bridging the gap in existing literature and informing policy efforts aimed at improving the educational success of multicultural students.

In light of the aforementioned challenges, this study examines the impact of acculturative stress on the achievement motivation of multicultural students in South Korea, focusing on the moderating role of mothers’ Korean language proficiency. Although previous research has examined the individual effects of acculturative stress and maternal language ability, less attention has been paid to how these two factors might interact. We posit that maternal Korean proficiency may moderate the relationship between acculturative stress and achievement motivation, such that the negative impact of stress is mitigated when mothers have strong language proficiency. While Park et al. (2023) primarily tested the mediating role of acculturative stress in the relationship between maternal Korean proficiency and children’s psychological well-being, their findings provide indirect support for the potential buffering role of maternal language ability. Given that maternal proficiency has been shown to reduce acculturative stress, which is an established negative predictor of various motivational and psychological outcomes, it is plausible to hypothesize that maternal language proficiency may also attenuate the adverse effects of acculturative stress on achievement motivation. In this context, maternal proficiency can serve as a protective factor, facilitating communication with schools, enabling access to institutional resources, and providing more effective emotional and academic support within the family.

By investigating these relationships, the study aims to offer empirical evidence to inform educational policies and intervention strategies that support the integration and success of students from multicultural families. Furthermore, this study addresses a critical gap by providing empirical validation of the effectiveness of Korean language education programs as part of multicultural support initiatives. By highlighting the dynamic interaction between acculturative stress and the linguistic environment in multicultural families, the study underscores the need for comprehensive support systems addressing both educational disparities and family dynamics. Finally, the findings are expected to offer valuable insights for designing interventions and policies to promote the academic and social success of multicultural students in South Korea.

### Research aims

This study investigates the impact of acculturative stress on the achievement motivation of multicultural students in South Korea and aims to address the following research questions:

1. How does acculturative stress negatively affect the achievement motivation of multicultural students in South Korea?
2. How does immigrant mothers’ high proficiency in Korean moderate the relationship between acculturative stress and achievement motivation?

### Multicultural students in South Korea

According to the Multicultural Families Support Act of South Korea, multicultural students in South Korea are defined as children from families in which one or both parents are foreign nationals or naturalized citizens. Unlike countries such as the United States, where multiculturalism is often embraced as part of a broader societal identity, South Korea has traditionally maintained a mono-ethnic and culturally homogeneous perspective. As a result, multicultural students in South Korea frequently face unique challenges, including language barriers, cultural adaptation, and social stigmatization (Kim and Kim, 2012; Kim and Seo, 2021; Lee et al., 2012). While multicultural students from immigrant backgrounds in the U.S. may benefit from established systems for bilingual education and cultural inclusion, those in South Korea often encounter limited institutional resources, with a heavy emphasis on Korean language proficiency as a prerequisite for integration (Hwang, 2018).

This policy-driven understanding of “multicultural” stands in contrast to how similar family structures are conceptualized in Western immigration literature. In those contexts, terms such as “mixed-nativity” or “cross-nativity” are commonly used to describe families in which the parents differ in nativity status, for example, one is native-born and the other is foreign-born. These terms are used to explore intergenerational differences, family dynamics, and assimilation trajectories (Pham and Altman, 2024). While conceptually related, the Korean term “multicultural” reflects state-led efforts to promote social cohesion and linguistic integration, rather than focusing solely on demographic distinctions or citizenship status. This distinction underscores the unique sociopolitical context in which multicultural students are situated in South Korea.

While the number of multicultural students has continued to grow, particularly in rural regions where international marriages are more prevalent, the country’s support system has struggled to keep pace. Various initiatives have been introduced, multicultural family support centers, Korean language programs, and after-school services aimed at promoting students’ academic and social development. However, significant gaps remain in addressing systemic issues, such as the cultural biases in the education system and the lack of comprehensive support for dual cultural identities (Oh, 2018). This underscores the need to explore how factors like acculturative stress and mothers’ Korean language proficiency influence multicultural students’ achievement motivation, thereby informing more effective policies and interventions to support their integration and success.

## Methods

### Participants

Data from the Multicultural Adolescents Panel Survey (MAPS), launched in 2011, was employed in this study. This ongoing longitudinal follow-up study is conducted annually by the National Youth Policy Institute (NYPI) in South Korea. The primary aim of MAPS is to understand the developmental characteristics of students from multicultural families in South Korea and to provide tailored policy support for this demographic. The survey began with students in the fourth grade (approximately 9 to 10 years old) and is scheduled to continue through 2025, when participants will be 23 or 24 years old. This study specifically utilized data from the 6th wave of the survey, conducted in 2016, when participants were in the ninth-grade, the final year of compulsory education in South Korea. This point in the educational trajectory is significant, as students begin to make critical decisions about their future schooling, such as high school placement. Given the developmental importance of this transitional period, ninth-grade was selected as the focal point for analyzing students’ academic motivation.

MAPS data are collected by NYPI in collaboration with local schools, using a stratified sampling framework based on school size and region. Participants were recruited through school administrators, with informed consent obtained from both students and their caregivers. The initial panel consisted of 4,452 fourth-grade students from multicultural families across 2,537 elementary schools nationwide. The sampling procedure was conducted in two stages: schools were first selected using stratified random sampling by region, and then probability proportional to size (PPS) sampling was applied to increase the likelihood of selecting schools with a larger number of multicultural students, thereby ensuring a self-weighted sample. Within each selected school, all eligible students were included in the study in principle. Based on statistical precision and available resources, the target sample was set at 1,600 adolescents and their respective parents, with an overall sampling rate of 35.9%. While the sampling strategy ensured broad national coverage, it was not designed to match the exact demographic distribution of multicultural families by maternal nationality. This characteristic of the sample should be considered when interpreting the contextual boundaries of the findings.

The survey is primarily conducted through Computer-Assisted Personal Interviewing (CAPI), although Paper-and-Pencil Interviewing (PAPI) was used in households that declined CAPI. Retention efforts, including regular follow-up contacts and small incentives, have contributed to maintaining a relatively stable sample across survey waves. Although some attrition occurred due to school transfers, relocation, or withdrawal of consent, the attrition rate remained low. According to NYPI documentation, data processing involves systematic cleaning procedures that address input errors, missing values, and logical inconsistencies. Given the paired nature of data collection from both students and their parents, NYPI conducts both cross-sectional and longitudinal data cleaning, linking student, household, and parental records across waves. Although specific imputation methods are not detailed in public documentation, the released data reflect robust quality control and validation procedures that enhance data reliability and usability.

A total of 1,329 students participated in the 6th wave of the survey. Forty-two students with Korean mothers were excluded from the analysis due to the focus on maternal Korean language proficiency as a key variable. Consequently, the final analytic sample consisted of 1,287 students, including 635 boys (49.3%) and 652 girls (50.7%). The demographic composition of the sample included 456 (35.4%) students whose mothers were Japanese, 343 (26.7%) with Filipina mothers, 336 (26.1%) with Chinese (including Korean Chinese) mothers, 51 (4.0%) with Thai mothers, 34 (2.6%) with Vietnamese mothers, and 67 (5.2%) with mothers from other countries. Most participants, approximately 664 (51.6%), perceived their family’s socioeconomic status as difficult or extremely difficult. Additionally, 328 (25.5%) of the participants resided in major cities, 581 (45.1%) in small and medium-sized cities, and 378 (29.4%) in rural areas. This rigorous sampling and data collection methodology ensure the robustness and reliability of the findings, providing a comprehensive understanding of the targeted population.

### Measure

#### Achievement motivation

Achievement motivation was assessed using eight items adapted from Yu and Yang (1994), with modifications made to align the items with the South Korean learning context. A sample item includes, “I try my best to achieve the academic goals that I set.” Each item was rated on a 4-point Likert-type scale ranging from 1 (*not at all*) to 4 (*very much so*), with higher scores indicating a higher level of achievement motivation. The Cronbach’s alpha coefficient for the total scale was 0.89, demonstrating strong internal consistency.

#### Acculturative stress

In the national MAPS dataset, acculturative stress was assessed with a 10-item scale adapted from the Acculturative Stress Scale for International Students (ASSIS; Sandhu and Asrabadi, 1994) by the National Youth Policy Institute (NYPI). The items were primarily drawn from the Perceived Discrimination and Perceived Hate and Rejection subscales. They were systematically adapted by NYPI to the Korean school context by rephrasing items to reflect parental nativity and culture-specific experiences, omitting less salient items, and adding a small number of context-specific items (e.g., limited Korean proficiency). We employed the instrument in the form provided by NYPI, without additional modification or item reduction. Examples of these items include: “I do not want to attend school because my parents are not from Korea,” “I feel stressed living in Korea,” “I am stressed because of my limited proficiency in Korean,” “I feel pressured by those around me to conform to Korean cultural norms,” and “Korean people are causing distress to my family.” Each item was rated on a 4-point Likert-type scale (1 = *not at all*, 4 = *very much so*), with higher scores indicating greater acculturative stress. The Cronbach’s alpha coefficient for the total scale was 0.76, indicating acceptable internal consistency. The full Korean wording of the MAPS items cannot be reproduced in this manuscript, but it is available to qualified researchers upon request from NYPI.

#### Mothers’ Korean language proficiency

Immigrant mothers’ proficiency in speaking, writing, reading, and listening to Korean was assessed using items adapted from the Children of Immigrants Longitudinal Study (CILS) by Portes and Rumbaut (2018). The items were tailored to assess Korean language usage specifically in the context of South Korea, with responses rated on a 4-point Likert-type scale ranging from 1 (*not at all*) to 4 (*very much so*). Higher scores corresponded to greater proficiency in the Korean language. The Cronbach’s alpha coefficient for the total scale was 0.91, indicating excellent internal consistency.

### Control variables

We incorporated several control variables into our regression model to account for factors influencing acculturative stress, mothers’ Korean language proficiency, and achievement motivation. These variables include gender, perceived socioeconomic status, maternal age, maternal educational attainment, maternal employment status, maternal nationality, students’ region of residence, and parental marital status. Gender (Qin, 2006) and parental socioeconomic status (Hwang and Joo, 2022) have been identified as significant determinants of multicultural adolescents’ achievement motivation, while maternal demographic characteristics and marital status may affect mothers’ language proficiency and involvement with their children. Additionally, region of residence is associated with the social–emotional development of students from multicultural families (Han et al., 2014). These variables were carefully selected to ensure a robust analysis of the primary relationships in the study.

### Data analysis

The Statistical Package for the Social Sciences (SPSS) version 20.0 was employed to conduct the analysis. Missing values were less than 1.5% across all items used. Correlational coefficients among the variables, as well as means and standard deviations, were calculated to provide descriptive information about the data. Skewness and kurtosis were assessed to verify the assumption of normality. All key constructs, including achievement motivation, acculturative stress, and mothers’ Korean language proficiency, were measured using multiple items. Each variable was operationalized as a mean composite score of its respective items, and these composite scores were used in the regression analyses.

To examine the hypothesized interaction between acculturative stress and mothers’ Korean language proficiency on students’ achievement motivation, we conducted a hierarchical multiple regression (HMR) analysis. HMR was selected to control for confounding influences from students’ demographic and family background variables, allowing us to determine the unique contribution of the main theoretical predictors. By entering demographic controls in the first step and the theoretical variables in subsequent steps, this method provides a clear view of the added explanatory power (ΔR^2^) attributable to acculturative stress, mothers’ Korean proficiency, and their interaction.

In Step 1, the model included gender, perceived socioeconomic status, maternal age, maternal educational completion status, maternal employment status, maternal nationality, students’ region of residence, and parental marital status as control variables. In Steps 2 through 4, acculturative stress, mothers’ Korean language proficiency, and their interaction term were entered sequentially. Prior to creating the interaction term, the two continuous predictors were mean-centered to minimize multicollinearity (Aiken and West, 1991). When the interaction effect was significant, we plotted the graph using mothers’ Korean language proficiency values that were one standard deviation above (+1 *SD*) and below (−1 *SD*) the mean. We also tested the statistical significance of the regression coefficient for each slope (Aiken and West, 1991).

## Results

### Descriptive statistics and correlations

The results of the descriptive statistics and partial correlations in this study, as shown in Table 1, revealed significant relationships among students’ perceptions of acculturative stress, their mothers’ Korean language proficiency, and their own achievement motivation. Acculturative stress had a mean of 1.42 (*SD* = 0.33), while perceived mothers’ Korean language proficiency and students’ achievement motivation had means of 3.25 (*SD* = 0.55) and 3.06 (*SD* = 0.47), respectively. The skewness of the variables ranged from −0.02 to 1.40, while the kurtosis values ranged from −0.77 to 3.94. Since the absolute values of skewness and kurtosis were below 3.0 and 10.0 respectively, the data were assumed to follow a normal distribution (Kline, 2010). The partial correlation analysis, after controlling for the effects of gender, perceived socioeconomic status, maternal age, maternal educational completion status, maternal employment status, maternal nationality, students’ region of residence, and parental marital status, revealed that higher acculturative stress was significantly associated with lower perceived maternal Korean language proficiency (*r* = −0.14, *p* < 0.01) and lower achievement motivation (*r* = −0.18, *p* < 0.01). Conversely, higher perceived mothers’ Korean language proficiency was significantly related to higher achievement motivation (*r* = 0.20, *p* < 0.01). These findings highlight significant relationships among acculturative stress, perceived language proficiency, and achievement motivation, underscoring the potential interactive dynamics among these variables.

**Table 1:** Summary of partial correlations, means, and standard deviations for observed variables.

Variable		1	2	3
1.	Mothers’ Korean language proficiency	-		
2.	Acculturative stress	−0.14^**^	-	
3.	Achievement motivation	0.20^**^	−0.18^**^	-
	*M*	3.25	1.42	3.06
	*SD*	0.55	0.33	0.47
	Skewness	−0.02	1.40	−0.04
	Kurtosis	−0.77	3.94	0.63

*N* = 1,256. The variables for mothers’ Korean language proficiency, acculturative stress, and academic motivation were measured using a four-point response scale, ranging from 1 (not at all) to 4 (very much so). ^**^p < 0.01.

### Hierarchical regression analysis

We performed a hierarchical regression analysis to predict achievement motivation based on control variables, acculturative stress, and mothers’ Korean language proficiency. Table 2 provides the standardized regression coefficients (*β*s), R^2^, change in R^2^ for each model, and 95% confidence intervals. When the interaction effect was significant, an interaction graph was plotted to illustrate the moderation effect. This graph depicted mothers’ Korean language proficiency at one standard deviation above the mean (+1 *SD*) and one standard deviation below the mean (−1 *SD*).

**Table 2:** Hierarchical multiple regression analysis examining interaction between acculturative stress and mothers’ Korean language proficiency on achievement motivation.

Predictor	Achievement motivation				
	B	β	ΔR^2^	95% CI	
				LL	UL
Step 1			0.026^***^		
Gender	0.009	0.01		−0.042	0.061
Perceived socioeconomic status	0.042	0.066^*^		0.006	0.077
Maternal age	−0.003	−0.034		−0.008	0.002
Maternal educational completion status	0.075	0.143^***^		0.044	0.105
Maternal employment status	0.004	0.028		−0.004	0.011
Maternal nationality	−0.012	−0.041			
				−0.030	0.005
Students’ region of residence	−0.006	−0.009			
				−0.041	0.029
Parental marital status	−0.021	−0.028		−0.062	0.021
Step 2			0.031^***^		
Acculturative stress	−0.259	−0.178^***^		−0.337	−0.180
Step 3			0.030^***^		
Mothers’ Korean language proficiency	0.156	0.183^***^		0.109	0.204
Step 4			0.004^*^		
Acculturative stress x Mothers’ Korean language proficiency	−0.160	−0.063^*^		−0.298	−0.021
Total *R^2^*	0.091^***^				
*N*	1,265				

^*^p < 0.05. ^**^p < 0.01. ^***^p < 0.001.

In Step 1, gender, perceived socioeconomic status, maternal age, maternal educational completion status, maternal employment status, maternal nationality, students’ region of residence, and parental marital status explained a significant portion of the variance in achievement motivation (Δ*R^2^* = 0.026, *p* < 0.001). Perceived socioeconomic status (*β* = 0.066, *p* < 0.05) and maternal educational completion status (*β* = 0.066, *p* < 0.05) were significant predictors of achievement motivation among students from multicultural backgrounds, while the remaining control variables were not significantly related. In Step 2, acculturative stress was added and accounted for a significant additional variance in achievement motivation (Δ*R^2^* = 0.031, *p* < 0.001), with a significant negative relationship to achievement motivation (*β* = −0.179, *p* < 0.001). In Step 3, mothers’ Korean language proficiency was included and significantly explained further variance in achievement motivation (Δ*R^2^* = 0.030, *p* < 0.001), showing a significant positive relationship to achievement motivation (*β* = 0.183, *p* < 0.001). The interaction between acculturative stress and mothers’ Korean language proficiency also significantly contributed to the variance explained (Δ*R^2^* = 0.004, *p* < 0.05).

**Figure 1:**
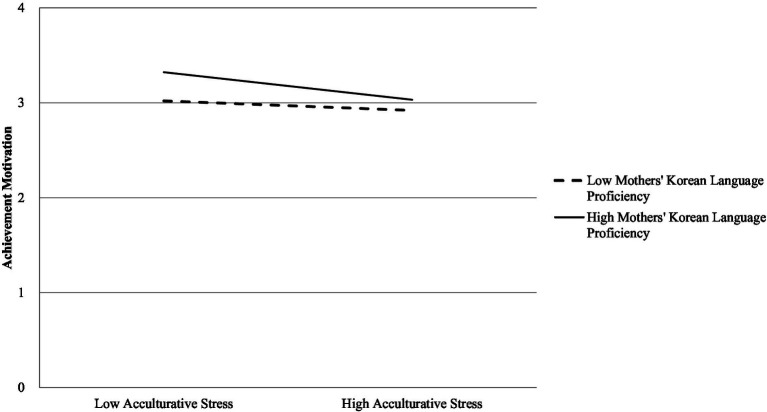
Relationship of acculturative stress with achievement motivation at different levels of mothers’ Korean language proficiency, *Note*. Solid line represents high maternal proficiency (+1 *SD*); dashed line represents low maternal proficiency (−1 *SD*).

Figure 1 illustrates the predictive pattern of acculturative stress on achievement motivation at different levels of mothers’ Korean language proficiency (±1 *SD*). The interaction graph illustrates that significant relationships between acculturative stress and achievement motivation were present for students with both high and low levels of mothers’ Korean language proficiency (*p* < 0.05). The graph indicates that students experience lower achievement motivation when they encounter high acculturative stress, and this decline is more pronounced when they perceive their mothers’ Korean language proficiency to be higher. Simple slope tests confirmed that the negative slope was steeper at high maternal proficiency, highlighting an exacerbation rather than a buffering effect.

## Discussion

This study analyzed data from the 6^th^ wave of the 2011 Multicultural Adolescents Panel Survey (MAPS) to examine the impact of acculturative stress and mothers’ Korean language proficiency on the achievement motivation of 9th grade multicultural students in South Korea. Additionally, the study investigated whether the Korean language proficiency of immigrant mothers moderates the effect of acculturative stress on achievement motivation. The findings provide critical insights into practical interventions to foster academic success and adaptation of multicultural students in South Korea.

This study confirmed that high levels of acculturative stress among students are significantly associated with low achievement motivation. This finding is consistent with previous research highlighting the role of mediating factors such as depression, social withdrawal, and self-esteem in linking acculturative stress to diminish achievement motivation (Lee et al., 2020; Park and Son, 2023). Moreover, these results reinforce existing evidence that acculturative stress adversely affects not only the mental health of students from multicultural backgrounds (e.g., Hovey, 2000) but also their academic performance. These findings emphasize the need for policymakers and educators to develop and implement targeted interventions that alleviate acculturative stress and foster academic success among multicultural students. For instance, the government could initiate nationwide research to investigate the underlying factors contributing to acculturative stress among multicultural students. At the same time, educators can cultivate an inclusive and non-discriminatory classroom environment that fosters a sense of belonging and psychological safety. Such efforts may help multicultural students develop a positive self-concept, thereby enhancing their academic motivation within the school context.

The relationship between acculturative stress and the achievement motivation of multicultural students differs significantly depending on their mothers’ Korean language proficiency. Students whose mothers have high Korean proficiency tend to exhibit higher achievement motivation when acculturative stress is low. However, as acculturative stress increases, their motivation declines more sharply compared to students whose mothers have low proficiency. In contrast, students with mothers of low Korean proficiency experience a more gradual decrease in achievement motivation as acculturative stress rises. These findings indicate that while higher maternal language proficiency is linked to greater achievement motivation under favorable conditions, it also seems to heighten students’ sensitivity to the negative effects of acculturative stress. In this sense, high maternal proficiency creates a vulnerability rather than a buffer, as achievement motivation begins from a higher baseline but drops more steeply under high stress. Thus, maternal proficiency functions less as a buffering factor and more as an exacerbating factor, accelerating the decline in achievement motivation under high stress.

These results partially align with previous Korean studies that emphasize the positive impact of immigrant mothers’ Korean language proficiency on their children’s development. For example, earlier research indicates that immigrant mothers’ proficiency in Korean is linked to greater involvement in their children’s school activities (Yu, 2020) and higher levels of self-esteem in their children (Park, 2010; Park, 2022), both of which significantly contribute to improved academic achievement (Lim, 2021). However, the present findings indicate that these advantages dissipate under high acculturative stress, revealing an exacerbation effect rather than a buffering effect.

This unexpected result could be attributed to developmental shifts in the roles of significant social agents in the lives of multicultural children, particularly in vulnerable situations. As children mature, peers often take on a more prominent role in fostering achievement motivation. Recent research supports this perspective, showing that peer support can mitigate the impact of acculturative stress on social withdrawal (Mo, 2018) and facilitate school adjustment by alleviating acculturative stress among multicultural students (Yu and Hwang, 2024). At the same time, the findings may also reflect the possibility that immigrant mothers’ fluency in Korean contributes to overprotective parenting behaviors (Jung, 2013). In such cases, immigrant mothers may become less influential as social agents, with their fluency in Korean potentially being perceived by their children as a source of stress, akin to excessive control or nagging. While these explanations are plausible, they remain speculative and should be interpreted with caution. Future qualitative research will be necessary to explore these underlying dynamics more fully. These findings point to a complex interaction between social influences and achievement motivation under high acculturative stress, underscoring the need for further research to explore these dynamics in the context of multicultural families.

In addition to building on these interpretations, our findings can also be understood through the lens of the acculturation gap-distress model, which emphasizes that conflicts may arise when parents and children adapt to the host culture at different rates (Telzer, 2010). Empirical evidence demonstrates that intergenerational cultural dissonance is associated with heightened parent–child conflict, weakened bonding, and subsequent problem behaviors among youth (Choi et al., 2008). In this regard, a mother’s relatively rapid acquisition of host language proficiency could be experienced by adolescents as an increased sense of cultural distance, potentially contributing to relational tension and challenges in school adjustment. From the perspective of self-determination theory (Deci and Ryan, 2000), such dynamics may place adolescents’ basic psychological needs for autonomy and competence at risk. For instance, when mothers assume more dominant roles in communication and social navigation, students may perceive a reduced sense of autonomy or competence, which could in turn weaken their motivation, particularly under stress. Related work on parental involvement suggests that discrepancies between parental orientations and adolescents’ developmental needs may hinder adjustment; for example, restrictive parental management of peer relationships in Mexican American families has been linked to less adaptive peer affiliations (Updegraff et al., 2010). Taken together, these studies highlight that mismatches in cultural adaptation or parental involvement, while not uniform in their effects, may contribute to tensions that challenge adolescents’ well-being and academic engagement.

These findings underscore the need to reconsider the scope of multicultural family support services provided by the Ministry of Gender Equality and Family in South Korea. While initiatives such as free Korean language courses for immigrant mothers have been effective, particularly in enhancing parental involvement in education and promoting the academic success of multicultural students, there is an increasing need to expand these services to address more complex and multifaceted challenges. For example, multicultural family support centers could develop parent education programs aimed at strengthening immigrant mothers’ parenting self-efficacy. Specifically, psychological education initiatives could help mothers better understand the acculturation process and adopt effective strategies to support their children in managing elevated levels of acculturative stress. In addition, centers could offer integrated programs that actively engage both parents and children.

Crucially, given that high levels of Korean language proficiency among mothers do not mitigate the adverse impact of acculturative stress on children’s academic motivation, it is essential to move beyond individual-level support. Interventions should also address the relational dynamics within the parent–child dyad. In this regard, interaction-focused programs, such as family counselling designed to promote mutual understanding and communication, may be particularly effective in fostering the academic adjustment of multicultural students. Notably, although educational and career counselling services are available at the local government level for multicultural students, recent research indicates that these services are not particularly effective for ninth-grade students (Ahn et al., 2024). This suggests that current programs do not adequately address the psychological stress and family dynamics that are critical for successful adaptation. Therefore, based on the findings of this study, it is imperative to diversify and tailor multicultural family support services to reflect the developmental stages and contextual needs of all family members, thereby ensuring more comprehensive and effective support for adaptation and academic success.

Despite its contribution, this study has several limitations. First, it relied on students’ perceptions of their mothers’ Korean language fluency as a proxy for actual proficiency. Although such perception-based measures reflect students’ lived experiences, they are susceptible to reporting bias and may diverge from objective assessments. In particular, adolescents experiencing higher levels of acculturative stress may underestimate parental support or misjudge maternal fluency, which could bias the observed moderation effect. At the same time, perception based ratings are widely used in developmental and acculturation research because they capture the adolescent’s subjective reality, which is often the most proximal driver of motivation and adjustment (e.g., Choi et al., 2008; Telzer, 2010). Future studies may benefit from incorporating both self-report and direct proficiency measures to enhance construct validity. Second, participants came from various multicultural backgrounds based on maternal nationality, but the present analysis did not fully capture subgroup-specific differences. Cultural, linguistic, and migration-related dynamics may influence how acculturative stress and academic motivation manifest across different ethnic groups. Future research should incorporate subgroup analyses to better account for this heterogeneity.

Third, although the MAPS dataset was constructed using stratified and probability proportional to size (PPS) sampling methods, the design aimed to increase multicultural representation rather than mirror national demographic proportions. As a result, certain groups such as students with Japanese or Filipina mothers are overrepresented, while others (e.g., Vietnamese or Central Asian families) are underrepresented. This sampling imbalance may affect the generalizability of findings to Korea’s broader multicultural youth population. Future research could mitigate this issue through weighted modeling or more inclusive sampling approaches. Fourth, achievement motivation tended to be domain-specific, meaning that students may exhibit varying levels of motivation depending on the subject area (e.g., mathematics, language arts) or task type (e.g., academic vs. extracurricular activities). This study, however, employed a domain-general measure, which may not fully capture such contextual differences.

Fifth, social context may shape the main effects. For example, multicultural youth from regions with higher proportions of multicultural populations, or from schools with stronger academic performance, may exhibit different patterns compared to those in less favorable contexts. Although this study included control variables to address contextual influences, they were insufficient to fully capture these effects. Future research should account for the clustered nature of multicultural youth data, either by testing moderation effects of social context or by applying hierarchical linear modelling (HLM), which may uncover more nuanced interactions.

Sixth, previous studies have identified gender differences in achievement motivation (e.g., Maheswari and Aruna, 2016; Shekhar and Devi, 2012). Although the present study treated gender as a control variable to account for its effects, future research should examine potential interactions between gender and the variables of interest. Finally, because some acculturative stress items are sensitive (e.g., school avoidance attributed to parent nativity), responses may be affected by social desirability bias despite standardized administration and confidentiality assurances. This possibility should be taken into account when interpreting self-reported stress levels. Therefore, caution is warranted in interpreting the results, and future studies should consider using domain-specific instruments to provide more nuanced insights.

In this study, we examined the relationship between acculturative stress and achievement motivation, particularly focusing on the role of mothers’ Korean language proficiency for multicultural students in South Korea. To our knowledge, this is the first study to explore the dual effects of mothers’ Korean language proficiency and its critical role in the development of multicultural students. The results indicate that multicultural students interpret their mothers’ proficiency differently depending on their levels of acculturative stress. When students experience low levels of stress in adjusting to different cultures, they perceive their mothers’ language proficiency as beneficial for enhancing their achievement motivation. In contrast, when they experience high levels of stress, they perceive their mothers’ language proficiency as an additional burden. The findings indicate that in vulnerable situations, protective factors such as mothers’ Korean proficiency may not sufficiently buffer against academic challenges. This points to the need for further research examining the conditional effectiveness of such factors.

## Data Availability

Publicly available datasets were analyzed in this study. This data can be found at: https://www.nypi.re.kr/archive/board?menuId=MENU00221. Further inquiries can be directed to the corresponding author.
